# The role of miR-19b in the inhibition of endothelial cell apoptosis and its relationship with coronary artery disease

**DOI:** 10.1038/srep15132

**Published:** 2015-10-13

**Authors:** Yong Tang, Ya-chen Zhang, Yu Chen, Yin Xiang, Cheng-xing Shen, Yi-gang Li

**Affiliations:** 1 Department of Cardiology, Xinhua Hospital, Shanghai Jiaotong University School of Medicine, Shanghai, China

## Abstract

The biological effects of microRNAs (miRNAs) and TNF-α in atherosclerosis have been widely studied. The circulating miR-17-92 cluster has been recently shown to be significantly downregulated in patients with injured vascular endothelium. However, it remains unclear whether the miR-17-92 cluster plays a significant role in vascular endothelial repair. The aim of this study was to investigate the relationship between the miR-17-92 cluster and TNF-α-induced endothelial cell apoptosis. We determined that the down-regulation of miR-19b level among patients with coronary artery disease was consistent with miRNA expression changes in endothelial cells following 24 h of TNF-α treatment. *In vitro*, the overexpression of miR-19b significantly alleviated the endothelial cells apoptosis, whereas the inhibition of miR-19b significantly enhanced apoptosis. The increased levels of Afap1 and caspase7 observed in our apoptosis model could be reduced by miR-19b, and this effect could be due to miR-19b binding 3′-UTRs of Afap1 and caspase7 mRNA. Therefore our results indicate that miR-19b plays a key role in the attenuation of TNF-α-induced endothelial cell apoptosis and that this function is closely linked to the Apaf1/caspase-dependent pathway.

Inflammatory factors can destroy the integrity of the endothelium, triggering the development of atherosclerosis. Tumor necrosis factor-α (TNF-α), an important inflammatory factor, induces endothelial cell injury, which results in endothelial dysfunction^1^,^2^. The caspase family members, including initiator caspases (e.g., caspase-8, -9, and -10) and effector caspases (e.g., caspase-3 and -7), are essential to the apoptotic process^3^. Numerous risk factors related to coronary artery diseases (CAD) contribute to increases in circulating TNF-α concentrations, resulting in higher caspase expression^3^. Therefore, reducing caspase levels may be helpful in attenuating both the development and the progression of CAD.

MicroRNAs (miRNAs), which consist of 18 to 24 nucleotides, can negatively regulate gene expression by binding to sites in the 3′ untranslated region (3′-UTR) of a target mRNA. Approximately 1000 miRNAs have been identified in humans^4^. Each miRNA can regulate hundreds of target mRNAs, as well as nearly one-third of proteins^4^,^5^. As a result, the biological effects of miRNAs are extensive and include cell growth, differentiation, and apoptosis.

In recent years, circulating miRNAs have been widely recognized as biomarkers of coronary heart disease^6^,^7^. Researchers have noted that the miR-17-92 cluster is significantly downregulated among patients with atherosclerosis^8^. The miR-17-92 cluster, which includes miR-17, miR-18a, miR-19a/b, miR-20a and miR-92a, has been extensively studied in different types of cancers^9^,^10^ (Table 1). Research has also demonstrated that miR-19 and miR-92 act to maintain an intricate balance between the pathways that promote and suppress cancer^11^. miR-17 and miR-20a influence cellular proliferation via the E2F family of transcription factors^12^. Additionally, miR-17-92 is highly expressed in normal human endothelial cells^13^. Researchers have noted that inhibiting miR-92a results in enhanced blood vessel growth and the functional recovery of damaged tissues^13^. miR-17/20 exhibits cell-intrinsic anti-angiogenic activity in endothelial cells^14^. These findings suggest that a specific miR-17-92 family member may be involved in the development of cardiovascular disease. However, whether the miR-17-92 cluster regulates apoptotic gene expression in endothelial cells needs to be further studied.

PTEN (a phosphatase and tensin homolog deleted from chromosome 10) is regulated by TNF-α and plays a crucial role in apoptosis^15^,^16^. Additionally, PTEN is a target gene of miR-17-92^17^, which suggestes that PTEN contributes to TNF-α induced apoptosis in endothelial cells. However, it remains unclear whether the miR-17-92 cluster is associated with endothelial dysfunction; therefore, the aim of this study was to analyze the changes in the levels of the miR-17-92 cluster in clinical patient sample and determine the role that the miR-17-92 cluster may play in various signaling pathways associated with TNF-α-induced endothelial cell apoptosis.

## Results

### Apoptosis of HUVECs is induced by TNF-α

**Figure 1:**
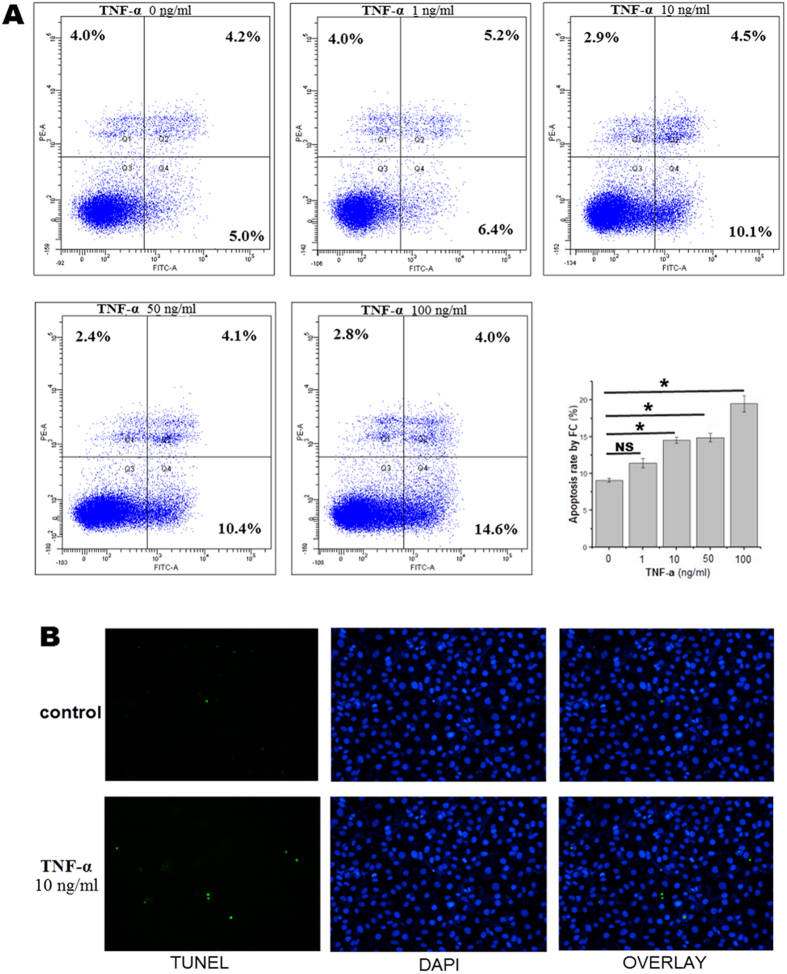
Effects of TNF-a on HUVEC apoptosis. Cells were treated with 0, 1, 10, 50 or 100 ng/ml of TNF-α for 24 hours. (**A**) Cells were stained with FITC Annexin V and propidium iodide (PI) for flow cytometry (FC) analysis. (**B**) Photomicrographs of the TUNEL –stained, DAPI-stained and an overlay of both TUNEL and DAPI stained cells treated with different concentrations of TNF-α. *p <0.05.

Apoptosis of endothelial cells treated with 0, 1, 10, 50 or 100 ng/ml of TNF-α for 24 h was measured by flow cytometry (Fig. 1A) and TUNEL/DAPI-stained cell photomicrography (Fig. 1B). The two methods demonstrated that TNF-α induces endothelial cell apoptosis in a dose-dependent manner, and that the lowest effective dose of 24 h TNF-α treatment was 10 ng/ml.

### Changes in miRNA expression in TNF-α-treated HUVECs

**Figure 2:**
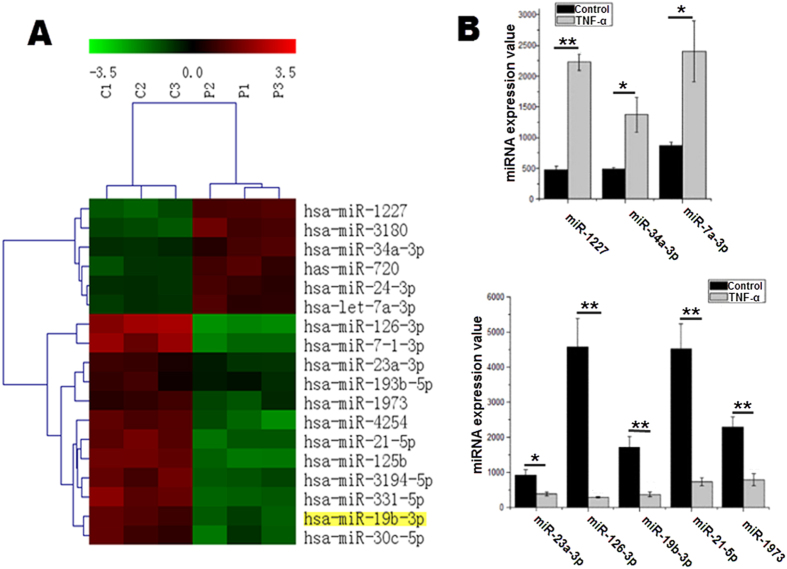
Changes in miRNA expression in TNF-a-treated (10 ng/ml, P1 P2 P3) or vehicle-treated (C1 C2 C3) HUVECs. (**A**) The log2 value of each miRNA signal was displayed on a heat map generated using Cluster 3.0 software. Red represents upregulation, green represents downregulation and black represents no change. The top bar code represents the color scale of the log2 values. (**B**) The signal values of the three upregulated miRNAs. (**C**) The signal values of the five downregulated miRNAs. *p <0.05 **p < 0.01.

Recent studies have demonstrated that dozens of miRNAs are highly expressed in normal human umbilical vein endothelial cells (HUVECs)^13^,^18^. However, little is known about which miRNAs play a key role in TNF-α-induced endothelial cell apoptosis. Accordingly, we used a microarray chip to analyze changes in miRNA expression in endothelial cells in an apoptosis model. The heat map revealed that 18 miRNAs (6 of which were upregulated and 12 of which were downregulated) exhibited a significant change compared with the control group (Fig. 2). The real-time PCR results were consistent with the microarray chip results. In the miR-17-92 cluster, miR-19b, which has a relatively high expression level in normal endothelial cells, was significantly downregulated following 24 h of TNF-α treatment. The overexpression of miR-19b did not influence the expression of other miRNAs, which exhibited expression patterns similar to that of miR-19b induced by TNF-α treatment (Supplementary Fig. S1).

### Apaf1 and Casp7 are target genes of miR-19b

**Figure 3:**
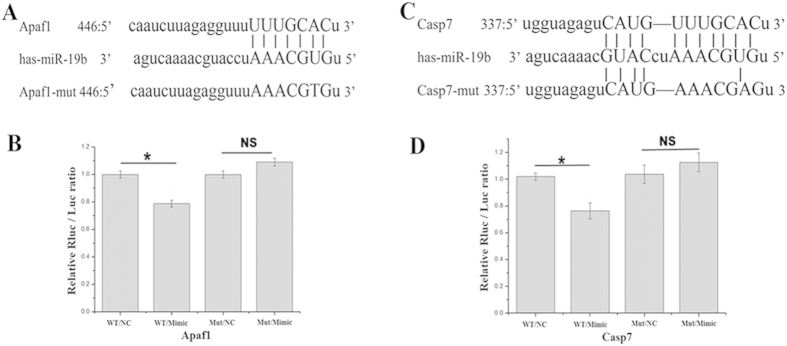
(**A,C**) Wild type and mutant binding sites for miR-19b in the 3′-UTR of Apaf1 or Casp7. (**B,D**) The relative luciferase value of the interaction between miR-19b and the 3′-UTR of Apaf1 or Casp7. WT, wild type; NC, negative control; Mut, mutant. *p < 0.05.

According to several publicly available bioinformatics web sites (TargetScan, miRanda and miRBase), both Apaf1 and Casp7 may be direct targets of miR-19b in endothelial cells. The 3′-UTRs of Apaf1 and Casp7 mRNA have binding sites for miR-19b (Fig. 3). The minimum free energy values of miR-19b-Apaf1 and Casp7 hybridization via RNAhybrid software were both −20.9 kcal/mol. To verify this finding, the 3′-UTRs of these potential target genes were cloned into luciferase reporter plasmids. miR-19b significantly the decreased luciferase activity of the wild type reporter plasmids, but no suppression of activity was observed with respect to the mutant reporter plasmids. In conclusion, these results demonstrated that both Afap1 and Casp7 are target genes of miR-19b.

### miR-19b modulates both Apaf1 and Casp7 expression in TNF-α-treated HUVECs

**Figure 4:**
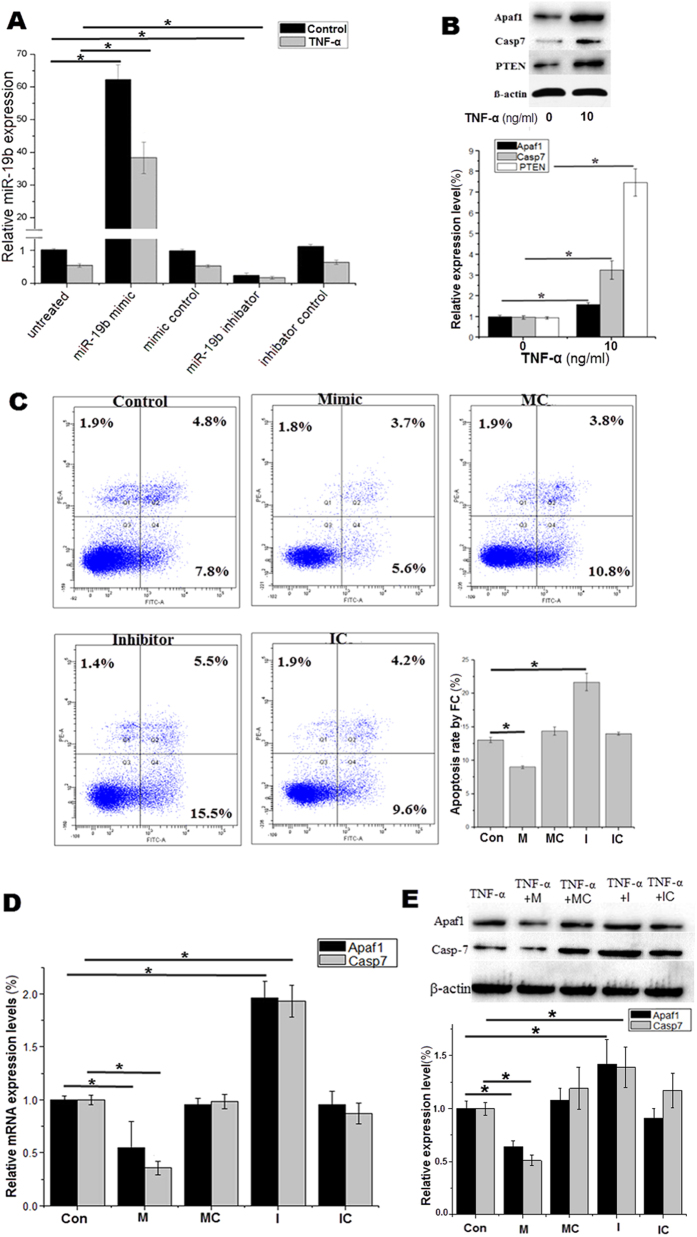
Effects of miR-19b on TNF-α-induced HUVEC apoptosis. (**A**) Changes in miRNA-19b levels of the HUVECs at 24 h following transfection with the vehicle, the miR-19b mimic, the mimic control, the miR-19b inhibitor or the inhibitor control under different conditions (in the presence or absence of TNF-α at 10 ng/ml). (**B**) Protein levels of Apaf1, Casp7 and PTEN in endothelial cells increased following 24 h of TNF-α (10 ng/ml) treatment. (**C**) Cells were stained with FITC Annexin V and PI for FC analysis. (**D,E**) The miR-19b mimic decreased both the mRNA and protein expression levels of Apaf1 and Casp7, whereas the miR-19b inhibitor increased the mRNA and protein levels of these proteins. *Mimic* miR-19b mimic; *MC* mimic control; *Inhibitor* miR-19b inhibitor; *IC* inhibitor control. *p < 0.05 **p < 0.01. The full-length blots are presented in Supplementary Figs S3 and S4.

An miR-19b mimic and inhibitor were each transfected into endothelial cells (Fig. 4A). The miR-19b mimic upregulated, whereas the miR-19b inhibitor downregulated miR-19b expression in a dose-dependent manner. RT-PCR and fluorescence microscopy demonstrated a relatively high transfection efficiency (approximately 90%) of both the mimic and the inhibitor at 50 nM concentration into HUVECs. By contrast, the oligo controls had no effect on miR-19b levels, either at high or low concentrations (data not shown). The protein levels of Apaf1, Casp7 and PTEN in the endothelial cells increased following 24 h of TNF-α treatment (Fig. 4B). We attempted to investigate the role of miR-19b in TNF-α-induced apoptosis by over- and under-expressing miR-19b in endothelial cells. After 24 h, flow cytometry demonstrated that the miR-19b mimic significantly alleviated apoptosis (12.6% vs 9.3%), whereas the miR-19b inhibitor significantly enhanced apoptosis (12.6% vs 21.0%, Fig. 4C). The miR-19b mimics, which upregulate miR-19b expression, significantly reduced the expression levels of Apaf1 (by approximately 30%) and Casp7 (by approximately 70%). However, the miR-19b inhibitors, which decrease miR-19b expression, increased the expression levels of both Apaf1 (by approximately 20%) and Casp7 (by approximately 2.5 times) (Fig. 4D,E). Therefore, miR-19b might directly regulate both Apaf1 and Casp7 expression at the post-transcriptional level in HUVECs.

### Effects of Apaf1-siRNA, Casp3-siRNA, Casp7-siRNA and PTEN-siRNA on TNF-a-induced apoptosis in HUVECs

**Figure 5:**
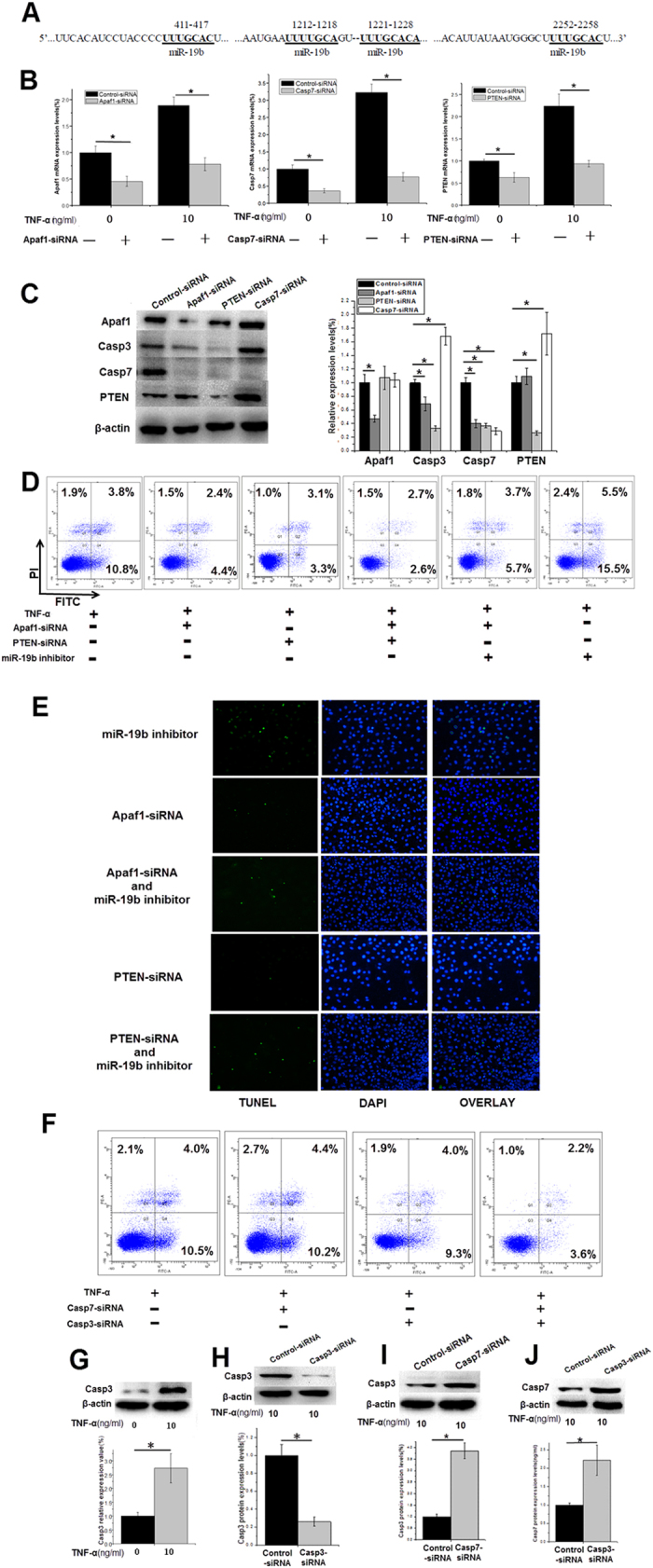
Effects of Apaf1-siRNA, PTEN-siRNA and miR-19b on TNF-a-induced HUVECs apoptosis. (**A**) The binding sites for miR-19b in the 3′-UTR of PTEN. (**B**) Effects of the knockdown of Apaf1, PTEN or Casp7 by specific siRNA on the mRNA levels. (**C**) Relationship between the Apaf-1/caspase pathway and the PTEN signaling pathway. (**D**) Cells transfected with siRNA or the miR-19b inhibitor were stained with FITC Annexin V and PI for FC analysis. (**E**) Cells transfected with siRNA/miR-19b inhibitor were stained by TUNEL. (**F**) Apoptosis after transfecting Casp7-siRNA and Casp3-siRNA. (**G**) Protein levels of Casp3 in the endothelial cells increased following 24 h of TNF-α (10 ng/ml) treatment. (**H**) Effects of the knockdown of Casp3 by specific siRNA on the mRNA and protein levels. (**I)** Casp3 levels following the downregulation of Casp7. (**J**) The Casp7 levels following the downregulation of Casp3. *p< 0.05. The full-length blots are presented in Supplementary Figure S5 to S9.

The 3′-UTRs of PTEN mRNA have several binding sites for miR-19b (Fig. 5A). The tumor suppressor gene, PTEN induces apoptosis via the inhibition of PI3K-mediated signaling^19^. When cytochrome c binds to Apaf1, the complex recruits and activates Casp9. The apoptosome activates downstream effector caspases (Casp3 and Casp7), causing cell apoptosis^20^,^21^. We investigated the role of both PTEN and Apaf-1 in HUVECs apoptosis induced by TNF-α. We transfected HUVECs with Apaf1-siRNA, Casp7-siRNA and PTEN-siRNA for 24 h before adding TNF-α (10 ng/ml) for an additional 24 h. Compared with the negative control, the Apaf1-siRNA reduced Apaf1 levels by more than 50%; the Casp7-siRNA reduced Casp7 expression levels by more than 60%; and the PTEN-siRNA reduced PTEN levels by approximately 40% (Fig. 5B). The silencing effects of the Apaf1-siRNA inhibited both Casp3 (approximately 40% decreased) and Casp7 (approximately 55% decreased) protein expression, but did not influence the expression of PTEN. The silencing effects of PTEN-siRNA also decreased the levels of Casp7 (by approximately 55%) and Casp3 (by approximately 60%), whereas the expression of Apaf1 was not affected (Fig. 5C), which suggests that the Apaf1/caspase pathway functions independently of apoptosis. TNF-α induced HUVECs apoptosis was enhanced by the miR-19b inhibitor (Fig. 5D,E), which indicates that miR-19b regulates both Apaf1 and PTEN in TNF-α-induced HUVECs apoptosis. Furthermore, separate transfections with either Casp7-siRNA or Casp3-siRNA did not reduce TNF-α-induced apoptosis, whereas the combined transfection of the two siRNAs reduced apoptosis significantly (Fig. 5F). The protein expression levels of Casp3 in the endothelial cells increased approximately 2.5 times following 24 h of TNF-α treatment (Fig. 5G). The Casp3-siRNA decreased Casp3 protein expression levels by 65% (Fig. 5H). We also observed that the silencing of one increased the expression of the other in the TNF-α-treated HUVECs (Fig. 5I,J). This finding demonstrated that not only do Casp3 and Casp7 work together but also compete with each other^22^.

### The level of circulating miR-19b is lower in patients with CAD

**Figure 6:**
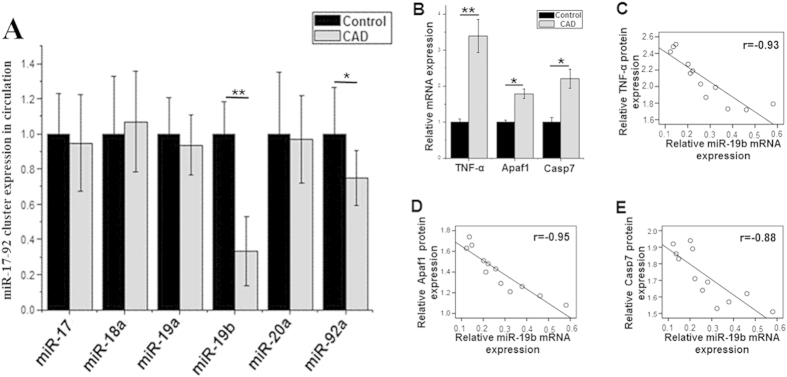
The levels of circulating miRNAs from the miR-17-92 cluster in healthy volunteers (n = 12) and patients with CAD (n = 12) (A). RT-PCR results demonstrate that the levels of TNF-α, Apaf1 and Casp7 mRNA were elevated in the specimens obtained from the patients with CAD (**B**). Spearman correlation coefficients revealed an inverse relationship between the levels of miR-19b and TNF-α, Apaf1 and Casp 7 protein in patients. (**C**) miR-19b and TNF-α, r = −0.93, p < 0.01; (**D**) miR-19b and Apaf1, r = −0.95, p < 0.01; (**E**) miR-19b and Casp 7, r = −0.88, p < 0.01) *p < 0.05 **p < 0.01.

The plasma levels of the miR-17-92 family of miRNAs were measured in twelve patients with CAD and twelve healthy subjects. There were no significant differences in the baseline characteristics of the two groups (Supplementary Table S1). Among the miR-17-92 family of miRNAs (Table 1), the levels of miR-19b and miR-92a were significantly lower in patients with CAD than in healthy subjects, as determined via RT-PCR (Fig. 6A). However, no differences were observed with respect to the levels of the other miRNAs between the two groups. These results indicated that a specific miRNA signature for CAD exists.

The RT-PCR results demonstrated that the expression levels of TNF-α, Apaf-1 and Casp7 were higher in the specimens obtained from the patients with CAD (Fig. 6B). The Spearman correlation coefficients revealed that there was an inverse relationship, at least in part, between the levels of miR-19b and TNF-α, Apaf1 and Casp7 protein in the patients analyzed (Fig. 6C–E) (miR-19b and TNF-α, r = −0.93, p < 0.01; miR-19b and Apaf1, r = −0.95, p < 0.01; miR-19b and Casp7, r = −0.88, p < 0.01).

## Discussion

In the pathological process of atherosclerosis, the inflammatory cytokine TNF-α plays a vital role in the disruption of the endothelial barrier and initiates apoptosis^1^,^2^,^23^. Numerous risk factors, such as advanced age, smoking, and over-nutrition, contribute to TNF-α concentration increases in the circulation. Elevated TNF-α exerts apoptotic effects via binding to TNF-α receptor type 1, which participates in caspase recruitment and activation^24^,^25^. The initiator caspases (e.g., caspase-8, -9, and -10) activate the effector caspases (e.g., caspase-3 and -7). The apoptosome which is composed of apoptotic protease-activating factor (Apaf-1), dATP, and procaspase-9, plays a key role in this progress. Apaf1 consists of three primary protein domains: a caspase recruitment domain, a nucleotide-binding oligomerization domain and multiple WD40 repeats. When cytochrome c binds to Apaf1, the complex recruits and activates caspase-9. The apoptosome activates the downstream effector caspases, triggering cell apoptosis^20^,^21^. Therefore, we selected TNF-α as the agent with which to induce endothelial cell apoptosis.

Several studies have reported that circulating miRNAs are linked to cardiovascular disease^6^,^7^,^26^,^27^. miR-1, miR-133, miR-208a and miR-499 levels are immediately upregulated in the plasma following an acute myocardial infarction^28^,^29^. Mature miR-423-5p molecules are strongly associated with the clinical diagnosis of heart failure^30^. As the first discovered oncogenic miRNA, the circulating miR-17-92 cluster is significantly downregulated in patients with coronary artery disease^8^. This finding was supported by our data, which demonstrated that within the miR-17-92 cluster, the levels of circulating miRNA-19b and miRNA-92a were significantly lower in patients with CAD compared with healthy control subjects. However, it remains unclear that how miRNAs are released into circulation. Accumulating evidence has demonstrated that circulating miRNAs are partially derived from microvesicles^31^. Therefore, it is necessary to understand role of the miR-17-92 cluster in atherosclerosis.

However, the effects and mechanisms underlying the effects of the miR-17-92 cluster on atherosclerosis remain unclear. As the miR-17-92 cluster is significantly downregulated in patients with CAD, and TNF-α is important with respect to the development of atherosclerosis, we hypothesized that the miR-17-92 cluster is closely associated with TNF-α-induced apoptosis in endothelial cells. Our miRNA microarray chip results supported this hypothesis. Following 24 h of TNF-α treatment at a concentration of 10 ng/ml, we observed a significant change in the expression of 18 miRNAs (including miR-19b) in the treated HUVECs compared with the control group. Furthermore, the miR-19b mimic upregulated miR-19b expression in the HUVECs and promoted cell survival, whereas the miR-19b inhibitor inhibited miR-19b expression and enhanced cell apoptosis. These results suggested that miR-19b plays a role in regulating the signaling pathways associated with TNF-α-induced HUVEC apoptosis.

**Figure 7:**
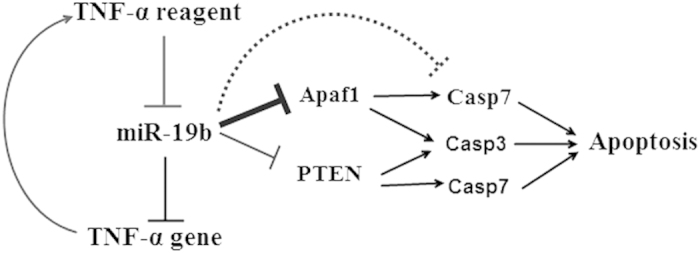
The mechanism diagram. Positive feedback was noted between miR-19b and TNF-α. miR-19b was significantly downregulated following 24 h of TNF-α treatment. The down-regulation of miR-19b alleviated its inhibition of TNF, which is the target gene of miR-19b, resulting in increased TNF-α expression. miR-19b plays a significant role in the attenuation of TNF-α-induced HUVEC apoptosis via the Apaf1/caspase pathway, which functions independently of apoptosis.

miRNAs function by binding to the 3′- UTRs of target mRNAs. In our study, the expression of miR-19b was lower in the TNF-α-treated endothelial cells, a finding accompanied by upregulation of Apaf1, Casp7, Casp3, and PTEN, which suggested that Apaf1/caspase and PTEN signaling pathway contribute to the anti-apoptotic effects of miR-19b in HUVECs treated with TNF-α. Then we tested the interaction between them (Fig. 5C), and concluded that miR-19b inhibits TNF-α-induced endothelial cell apoptosis via the Apaf1/caspase pathway, which is independent for apoptosis. Additionally, TNF was also a potential target gene of miR-19b^32^,^33^. The down-regulation of miR-19b alleviated its inhibition of TNF mRNA, resulting increased TNF-α expression, which subsequently resulted in reduced miR-19b expression. It appears that a form of positive feedback regulates this process (Fig. 7).

The miR-17-92 cluster contains 6 members containing a highly conserved 5′ seed region, including miR-17/20a (seed sequence: AAAGUG), miR-18a (AAGGUG), miR-19a/b (GUGCAA) and miR-92a (AUUGCA). The miR-17-92 family is highly expressed in human endothelial cells^13^; miR-19 is regarded as the primary oncogenic component of this family^34^,^35^. Olive V *et al.*^34^ observed that miR-19 promoted c-myc-induced lymphomagenesis by repressing apoptosis. In our study, we observed a significant change in miR-19b expression in HUVECs following 24 h of TNF-α treatment, as well as anti-apoptotic effects of miR-19b *in vitro*. However, miR-19a and miR-19b differ by only a single nucleotide at position 11 (Table 1). We therefore also studied the function of miR-19a with respect to the TNF-α-induced apoptosis of HUVECs and observed that miR-19a exereted similar anti-apoptotic effects (Supplementary Fig. S2). However, the reasons for the differences in the expression levels of miR-19a and miR-19b remain unclear. Although our research also demonstrated that the plasma levels of miR-92a were lower among patients with CAD than among healthy control subjects, Loyer X *et al.*^36^ recently observed that upregulation of miR-92a by ox-LDL in atheroprone areas promotes both endothelial cell activation and the development of atherosclerotic lesions. These findings indicated that miR-19 and miR-92a may exert opposing effects with respect to endothelial cell apoptosis. Regarding the other members of the miR-17-92 cluster, miR-17/20 exhibits cell-intrinsic anti-angiogenic activity in endothelial cells^14^, and miR-18a is the most strongly repressed miRNA in aged cardiomyocytes^37^.

Increasing evidence suggests that the biological functions of miRNAs may be either cell-type or microenvironment dependent. For example, miR-23a promotes apoptosis in human embryonic kidney cells^38^, but inhibits apoptosis in HUVECs^39^. The inhibition of miR-17/20a *in vivo* via antagomirs significantly increases the number of perfused vessels in Matrigel plugs, whereas the systemic inhibition of miR-17-20a does not affect tumor angiogenesis^14^. Accordingly, to minimize the effects of confounding variables, we selected patients with CAD and age/sex-matched healthy controls. We also used several different methods of testing the effects of the miRNAs *in vitro* to improve the reliability of our results. Although many research studies regarding atherosclerosis focus on HUVECs, the physiological characteristics of HUVECs are not completely consistent with those of coronary artery endothelial cells. The *in vivo* and *in vitro* environments are also different. Therefore, the effects of miR-19 with respect to endothelial cell apoptosis *in vivo* warrant further investigation. More studies are also necessary to clarify the *in vivo* effects exerted by the deletion of the miR-19 gene. Although more research is necessary, the findings of our study indicate that miR-19b exerts anti-apoptotic effects in endothelial cells, which may broaden the scope of the research regarding the role of miRNAs in atherosclerosis.

In conclusion, our findings demonstrated that miR-19b plays a significant role in the attenuation of TNF-α-induced HUVEC apoptosis, which is closely linked to the Apaf1/caspase-dependent pathway. These results could provide new insight into the clinical applications of endothelial repair in the setting of atherosclerosis.

## Methods

The experiments were conducted in accordance with approved guidelines: this study was approved by the ethics committee of Xinhua Hospital School of Medicine, Shanghai Jiaotong University, and informed consent was obtained from all patients before the study.

### Patient population and blood collection

Twelve patients diagnosed with CAD by our Division of Cardiology were recruited. Their diagnoses were based on a history of chest pain, coronary angiography results and characteristic ECG changes. In addition, twelve healthy subjects were enrolled to serve as a control group. Age, gender, BMI, and other baseline characteristics of the two groups were compared. A 10 ml sample of peripheral blood was collected in an EDTA-containing vacutainer tube from each individual for further analysis.

### Isolation and identification of HUVECs

Primary human umbilical vein endothelial cells (HUVECs) were cultured following a previously described protocol^40^. The cord, which was separated from the placenta, was stored in a sterile container filled with cord buffer. The blood in the umbilical vein was washed out using cord buffer. Ten milliliters of cord buffer containing 0.2% collagenase type II (Sigma, USA) was subsequently infused into the umbilical vein, which was clamped shut using hemostats. Following incubation at 37 °C for 15 min, the solution containing the endothelial cells was rinsed with an appropriate amount of cord buffer. The cells obtained from the solution were cultured in DMEM medium containing 10% fetal bovine serum and 1% penicillin–streptomycin at 37 °C in a 5% CO_2_ incubator. This entire operation used sterile techniques and was completed within three hours. Endothelial cells passages from three to five were used for this study.

### Flow cytometry and TUNEL staining analysis of cells treated with TNF-a

Endothelial cells were treated with 0, 1, 10, 50 or 100 ng/ml of TNF-α (Proptech) in 6-well plates for 24 hours. The effects of TNF-α on HUVEC apoptosis were evaluated by flow cytometry and TUNEL/DAPI-stained cell photomicrography. Flow cytometry was performed using a FITC Annexin V apoptosis detection kit I (BD). HUVECs were washed twice with cold PBS, resuspended in a 1X binding buffer, incubated in a 5 μl FITC-Annexin V solution for 10 min and then incubated with PI for 5 min at 25 °C in the dark. The cells were subsequently analyzed within 1 h before being analyzed a second time using a Beckman Coulter FC500 (Beckman). An *in situ* cell death detection kit (Roche) that involves TUNEL staining was used. TUNEL stained only the apoptotic cells, but when it was combined with DAPI, all of the cells were stained.

### miRNA microarray

To detect changes in miRNA expression in the TNF-α-treated HUVECs, a miRNA microarray was performed. HUVECs were treated with either vehicle (n = 3) or TNF-α (10 ng/ml) (n = 3) for 24 h. The total RNA from each group was isolated and hybridized using a microRNA microarray chip (Agilent). Locally weighted scatterplot smoothing (LOWESS) was utilized for background subtraction and signal normalization.

### Assessment of miRNA and mRNA levels

Total RNA was isolated using a RNA Isolation Kit (Ambion), and levels of the miR-17-92 cluster were measured using a mirVana qRT–PCR miRNA Detection Kit (Ambion). Reverse transcription reactions were specific for each miR-17-92 cluster miRNA, and U6 was used as the endogenous control. Expression levels of apoptotic protease-activating factor (Afap1) and caspase-7 (Casp7) were measured using a qRT-PCR mRNA detection kit (Roche) following the manufacturer’s instructions, ß-actin was used for normalization. The 2^_DDCt^ method was used to quantify the relative expression levels between the groups. The forward (F) and reverse (R) primers were as follows: has-miR-19b F: 5′- TGTTGCATGGATTTGCACA-3′, and R: 5′- GTGCAGGGTCCGAGGT-3′; Afap1 F: 5′- GATGATGTTTGGGACTCTTGG-3′, and R: 5′- AAGGAACTCTCCACAGGGACT-3′; Casp7 F: 5′- AGTGACAGGTATGGGCGTTC-3′, and R: 5′- GGCATTTGTATGGTCCTCT-3′; U6 F: 5′- CGCTTCGGCAGCACATATACTAAAATTGGAAC-3′, and R: 5′- GCTTCACGAATTTGCGTGTCATCCT-3′; β-actin F: 5′-AGAGCCTCGCCTTTGCCGAT-3′, and R: 5′-TGCCAGATTTTCTCCATGTCGT-3′.

### Assessment of protein levels

Western blots were performed in according to standard procedures. HUVECs were seeded in 6-well plates and lysed with 100 μl of a cell lysis solution containing 2 μl of phenylmethanesulfonyl fluoride buffer. Equal amounts of protein (40 μg) were loaded onto 12% SDS–PAGE gels (Millipore). Primary antibodies for Casp7 (1:1000 dilution; Cell Signaling), Casp3 (1:1000 dilution; Cell Signaling), Apaf1 (1:1000 dilution; Cell Signaling) and PTEN (1:1000 dilution; Cell Signaling) were used. The antibody for β-actin (1:1000 dilution; Cell Signaling) was used as the endogenous control. The gels were run under the same experimental conditions and the original whole gel blots were included in the supplementary data. The levels of TNF-α, Apaf-1 and Casp7 protein in the serum samples obtained from the patients with CAD were measured by Enzyme-Linked Immunosorbent Assay Kits (Elabscience).

### Transfection *in vitro*

miR-19b mimics and inhibitors (Dharmacon, Inc. 50 nM) were added to the culture media when the cells reached 50-60% confluence. The cells were transfected with the vehicle alone, an oligo control for the miR-19b mimic or an oligo control for the miR-19b inhibitor. Transfection efficiency was assessed using a GFP-labeled oligo control. Changes in miRNA expression were determined by qRT-PCR at 24 h following transfection. The Apaf1-siRNA, PTEN-siRNA, Casp3-siRNA and Casp7-siRNA were obtained from Santa Cruz Biotechnology.

### Luciferase activity assay

The 3′-UTR of Apaf1, which contains binding sites for miR-19b, was amplified by PCR using the following primers: F: 5′-GGCGGCTCGAGTAATGAGAAGAATTTGGAAGAAAT-3′; and R:5′ –AATGCGGCCGCCTAGACAACATAGTGAGACC-3′. We subsequently introduced point mutations at the seed binding sites of the 3′-UTR of Apaf1, using the following primers: F:5′ –AGAGGTTTAAACGTGTCTTTAAATTTGCTTTAA-3′; and R:5′- TTTAAAGACACGTTTAAACCTCTAAGATTGTAT-3′. Either the wild type or the mutant fragment was inserted into the pmiR-RB-REPORT^TM^ vector (Ribobio, China) at the same site. Either the miR-19b mimic or mimic control was subsequently transfected into the HEK 293 cells in the presence of either the wild type or the mutant reporter plasmid. Luciferase activity was measured at 24 h following transfection using the dual-Luciferase Reporter Assay System (Promega).

Similarly, either the wild type or the mutant 3′-UTR of Casp7 was transfected and evaluated using the same methods as described above. The primers of the wild type Casp7 were as follows: F: 5′ –GGCGGCTCGAGCCATATCAGGGGTACATTCT 3′, and R: 5′-AATGCGGCCGCAATTAAGCAA CCACATTTTT-3′. The primers of the mutant Casp7 were as follows: F: 5′-AGAGTCATGAAACGAGTTGG-3′, and R: 5′-CCAACTCGTTTCATGACTCT-3′.

### Statistical analysis

Statistical analysis was performed using SPSS 18 software (SPSS Inc., USA). For the normally distributed variables, comparisons between the two groups were performed using a t-test. The statistical significance of the variables in proportions was determined using the chi-squared test. The association between the levels of miR-19b and TNF-α, Apaf-1 and Casp7 was described using the Spearman correlation coefficient. A *P*-value < 0.05 was considered statistically significant.

## Additional Information

**How to cite this article**: Tang, Y. *et al.* The role of miR-19b in the inhibition of endothelial cell apoptosis and its relationship with coronary artery disease. *Sci. Rep.* **5**, 15132; doi: 10.1038/srep15132 (2015).

## Supplementary Material

Supplementary Information

## Figures and Tables

**Table 1:** Overview of the miR-17–92 cluster.

microRNA	Seed sequence	Mature miRNA sequence(5′—3′)
has-miR-17	AAAGUG	CAAAGUGCUUACAGUGCAGGUAG
has-miR-18a	AAGGUG	UAAGGUGCAUCUAGUGCAGAUAG
has-miR-19a	GUGCAA	UGUGCAAAUCUAUGCAAAACUGA
has-miR-19b	GUGCAA	UGUGCAAAUCCAUGCAAAACUGA
has-miR-20a	AAAGUG	UAAAGUGCUUAUAGUGCAGGUAG
has-miR-92a	AUUGCA	UAUUGCACUUGUCCCGGCCUGU

## References

[b1] Sullivan K. E. *et al.* Epigenetic regulation of tumor necrosis factor alpha. Mol Cell Biol 27, 5147–5160 (2007). 17515611 10.1128/MCB.02429-06 PMC1951949

[b2] Kleinbongard P., Heusch G. & Schulz R. TNFalpha in atherosclerosis, myocardial ischemia/reperfusion and heart failure. Pharmacol Ther 127, 295–314 (2010). 20621692 10.1016/j.pharmthera.2010.05.002

[b3] Cohen G. M. Caspases: the executioners of apoptosis. Biochem J 326(Pt 1), 1–16 (1997). 9337844 10.1042/bj3260001 PMC1218630

[b4] Bushati N. & Cohen S. M. microRNA functions. Annu Rev Cell Dev Biol 23, 175–205 (2007). 17506695 10.1146/annurev.cellbio.23.090506.123406

[b5] Adams B. D., Kasinski A. L. & Slack F. J. Aberrant regulation and function of microRNAs in cancer. Curr Biol 24, R762–R776 (2014). 25137592 10.1016/j.cub.2014.06.043 PMC4177046

[b6] Cheng C., Wang Q., You W., Chen M. & Xia J. MiRNAs as biomarkers of myocardial infarction: a meta-analysis. PLoS One 9, e88566 (2014). 24533109 10.1371/journal.pone.0088566 PMC3922900

[b7] Liebetrau C. *et al.* Release kinetics of circulating muscle-enriched microRNAs in patients undergoing transcoronary ablation of septal hypertrophy. J Am Coll Cardiol 62, 992–998 (2013). 23747779 10.1016/j.jacc.2013.05.025

[b8] Fichtlscherer S. *et al.* Circulating microRNAs in patients with coronary artery disease. Circ Res 107, 677–684 (2010). 20595655 10.1161/CIRCRESAHA.109.215566

[b9] Mogilyansky E. & Rigoutsos I. The miR-17/92 cluster: a comprehensive update on its genomics, genetics, functions and increasingly important and numerous roles in health and disease. Cell Death Differ 20, 1603–1614 (2013). 24212931 10.1038/cdd.2013.125 PMC3824591

[b10] Olive V., Li Q. & He L. mir-17-92: a polycistronic oncomir with pleiotropic functions. Immunol Rev 253, 158–166 (2013). 23550645 10.1111/imr.12054 PMC3972423

[b11] Olive V. *et al.* A component of the mir-17-92 polycistronic oncomir promotes oncogene-dependent apoptosis. Elife 2, e822 (2013). 10.7554/eLife.00822 PMC3796314 24137534

[b12] Pickering M. T., Stadler B. M. & Kowalik T. F. miR-17 and miR-20a temper an E2F1-induced G1 checkpoint to regulate cell cycle progression. Oncogene 28, 140–145 (2009). 18836483 10.1038/onc.2008.372 PMC2768269

[b13] Bonauer A. *et al.* MicroRNA-92a controls angiogenesis and functional recovery of ischemic tissues in mice. Science 324, 1710–1713 (2009). 19460962 10.1126/science.1174381

[b14] Doebele C. *et al.* Members of the microRNA-17-92 cluster exhibit a cell-intrinsic antiangiogenic function in endothelial cells. Blood 115, 4944–4950 (2010). 20299512 10.1182/blood-2010-01-264812

[b15] Takeda K. *et al.* BDNF protects human vascular endothelial cells from TNFalpha-induced apoptosis. Biochem Cell Biol 91, 341–349 (2013). 24032685 10.1139/bcb-2013-0005

[b16] Huang J. & Kontos C. D. PTEN modulates vascular endothelial growth factor-mediated signaling and angiogenic effects. J Biol Chem 277, 10760–10766 (2002). 11784722 10.1074/jbc.M110219200

[b17] Li X. *et al.* Curcumin modulates miR-19/PTEN/AKT/p53 axis to suppress bisphenol A-induced MCF-7 breast cancer cell proliferation. Phytother Res 28, 1553–1560 (2014). 24831732 10.1002/ptr.5167

[b18] Harris T. A., Yamakuchi M., Ferlito M., Mendell J. T. & Lowenstein C. J. MicroRNA-126 regulates endothelial expression of vascular cell adhesion molecule 1. Proc Natl Acad Sci USA 105, 1516–1521 (2008). 18227515 10.1073/pnas.0707493105 PMC2234176

[b19] Oudit G. Y. *et al.* The role of phosphoinositide-3 kinase and PTEN in cardiovascular physiology and disease. J Mol Cell Cardiol 37, 449–471 (2004). 15276015 10.1016/j.yjmcc.2004.05.015

[b20] Li P. *et al.* Cytochrome c and dATP-dependent formation of Apaf-1/caspase-9 complex initiates an apoptotic protease cascade. Cell 91, 479–489 (1997). 9390557 10.1016/s0092-8674(00)80434-1

[b21] Kim H. E., Du F., Fang M. & Wang X. Formation of apoptosome is initiated by cytochrome c-induced dATP hydrolysis and subsequent nucleotide exchange on Apaf-1. Proc Natl Acad Sci USA 102, 17545–17550 (2005). 16251271 10.1073/pnas.0507900102 PMC1266161

[b22] Brown M. F. *et al.* Loss of Caspase-3 sensitizes colon cancer cells to genotoxic stress via RIP1-dependent necrosis. Cell Death Dis 6, e1729 (2015). 25906152 10.1038/cddis.2015.104 PMC4650537

[b23] Zhang J., Patel J. M., Li Y. D. & Block E. R. Proinflammatory cytokines downregulate gene expression and activity of constitutive nitric oxide synthase in porcine pulmonary artery endothelial cells. Res Commun Mol Pathol Pharmacol 96, 71–87 (1997). 9178369

[b24] Dela P. N., Simeonidis S., Leo C., Rose D. W. & Collins T. Regulation of NF-kappaB-dependent gene expression by the POU domain transcription factor Oct-1. J Biol Chem 282, 8424–8434 (2007). 17192276 10.1074/jbc.M606923200

[b25] Rimbach G., Valacchi G., Canali R. & Virgili F. Macrophages stimulated with IFN-gamma activate NF-kappa B and induce MCP-1 gene expression in primary human endothelial cells. Mol Cell Biol Res Commun 3, 238–242 (2000). 10891398 10.1006/mcbr.2000.0219

[b26] Pilbrow A. P. *et al.* Circulating miR-323-3p and miR-652: candidate markers for the presence and progression of acute coronary syndromes. Int J Cardiol 176, 375–385 (2014). 25124998 10.1016/j.ijcard.2014.07.068

[b27] Wu J. *et al.* Identification of serum microRNAs for cardiovascular risk stratification in dyslipidemia subjects. Int J Cardiol 172, 232–234 (2014). 24461990 10.1016/j.ijcard.2013.12.214

[b28] Corsten M. F. *et al.* Circulating MicroRNA-208b and MicroRNA-499 reflect myocardial damage in cardiovascular disease. Circ Cardiovasc Genet 3, 499–506 (2010). 20921333 10.1161/CIRCGENETICS.110.957415

[b29] Kuwabara Y. *et al.* Increased microRNA-1 and microRNA-133a levels in serum of patients with cardiovascular disease indicate myocardial damage. Circ Cardiovasc Genet 4, 446–454 (2011). 21642241 10.1161/CIRCGENETICS.110.958975

[b30] Tijsen A. J. *et al.* MiR423-5p as a circulating biomarker for heart failure. Circ Res 106, 1035–1039 (2010). 20185794 10.1161/CIRCRESAHA.110.218297

[b31] Creemers E. E., Tijsen A. J. & Pinto Y. M. Circulating microRNAs: novel biomarkers and extracellular communicators in cardiovascular disease? Circ Res 110, 483–495 (2012). 22302755 10.1161/CIRCRESAHA.111.247452

[b32] Liu M. *et al.* TNF-alpha is a novel target of miR-19a. Int J Oncol 38, 1013–1022 (2011). 21271217 10.3892/ijo.2011.924

[b33] Ohira T. *et al.* miR-19b regulates hTERT mRNA expression through targeting PITX1 mRNA in melanoma cells. Scientific Reports 5, 8201 (2015). 25643913 10.1038/srep08201 PMC4314654

[b34] Olive V. *et al.* miR-19 is a key oncogenic component of mir-17-92. Genes Dev 23, 2839–2849 (2009). 20008935 10.1101/gad.1861409 PMC2800084

[b35] Mu P. *et al.* Genetic dissection of the miR-17~92 cluster of microRNAs in Myc-induced B-cell lymphomas. Genes Dev 23, 2806–2811 (2009). 20008931 10.1101/gad.1872909 PMC2800095

[b36] Loyer X. *et al.* Inhibition of microRNA-92a prevents endothelial dysfunction and atherosclerosis in mice. Circ Res 114, 434–443 (2014). 24255059 10.1161/CIRCRESAHA.114.302213

[b37] van Almen G. C. *et al.* MicroRNA-18 and microRNA-19 regulate CTGF and TSP-1 expression in age-related heart failure. Aging Cell 10, 769–779 (2011). 21501375 10.1111/j.1474-9726.2011.00714.x PMC3193380

[b38] Chhabra R., Adlakha Y. K., Hariharan M., Scaria V. & Saini N. Upregulation of miR-23a-27a-24-2 cluster induces caspase-dependent and -independent apoptosis in human embryonic kidney cells. PLoS One 4, e5848 (2009). 19513126 10.1371/journal.pone.0005848 PMC2689653

[b39] Ruan W., Xu J. M., Li S. B., Yuan L. Q. & Dai R. P. Effects of down-regulation of microRNA-23a on TNF-alpha-induced endothelial cell apoptosis through caspase-dependent pathways. Cardiovasc Res 93, 623–632 (2012). 22038739 10.1093/cvr/cvr290

[b40] Jaffe E. A., Nachman R. L., Becker C. G. & Minick C. R. Culture of human endothelial cells derived from umbilical veins. Identification by morphologic and immunologic criteria. J Clin Invest 52, 2745–2756 (1973). 4355998 10.1172/JCI107470 PMC302542

