# Supplementary material for: The role of miR-19b in the inhibition of endothelial cell apoptosis and its relationship with coronary artery disease
Source: Sci Rep. 2015 Oct 13;5:15132. doi: 10.1038/srep15132 (PMC4602285; doi:10.1038/srep15132)
Supplement: Supplementary Information [file srep15132-s1.pdf]

Supplementary Information file

Title: The role of miR-19b in the inhibition of endothelial cell apoptosis and its relationship with coronary artery disease

Authors: Yong Tang, Ya-chen Zhang*, Yu Chen, Yin Xiang, Cheng-xing Shen, Yi-gang Li

Supplementary Table S1 Characteristics of the patients

	Total	CAD group	Control group	*P*
N	24	12	12	
Age (year)		40.42±2.937	37.67±4,376	0.084
Male (n (%))		10(83.3)	11(91.7)	0.537
Body mass index, kg/m2		24.01±2.908	25.5±1.567	0.132
Hypertension (n (%))		5(41.7)	2(16.7)	0.178
Hyperlipidemia (n (%))		2(16.7)	1(8.3)	0.537
Diabetes (n (%))		4(33.3)	2(16.7)	0.346
Smoker (n (%))		7(58.3)	7(58.3)	1.000

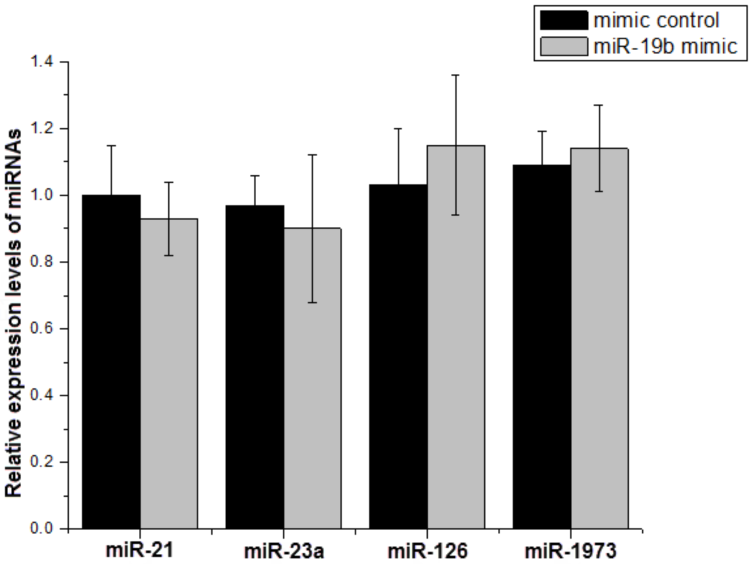

Supplementary Figure S1. The expression changes of miR-21, miR-23a, miR-126 and miR-1973 in HUVECs after transfection with the vehicle, the miR-19a mimic for 24h. Overexpression of miR-19b did not influence the expression of these miRNAs. $p<0.05$

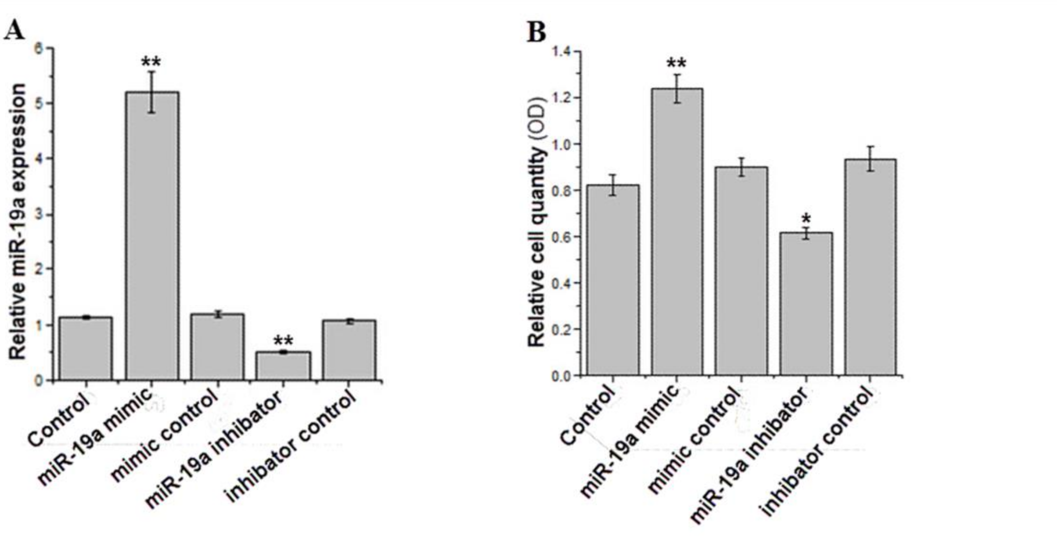

Supplementary Figure S2. The effects of miR-19a on TNF-α-induced HUVEC apoptosis. (A) Changes in miRNA-19a levels in HUVECs 24 h after transfection with the vehicle, the miR-19a mimic, the mimic control, the miR-19a inhibitor and inhibitor control. (B) Relative levels of cell proliferation were measured by the Cell Counting Kit-8. *$p<0.05$ **$p<0.01$

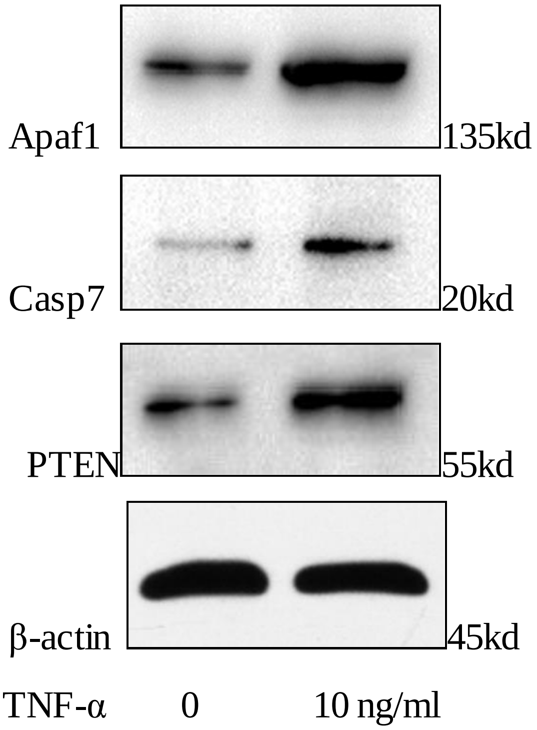

Supplementary Figure S3. Full length blots of data shown in Fig.4B

The gels were initially cut ranged from 15kd to 145 kd.

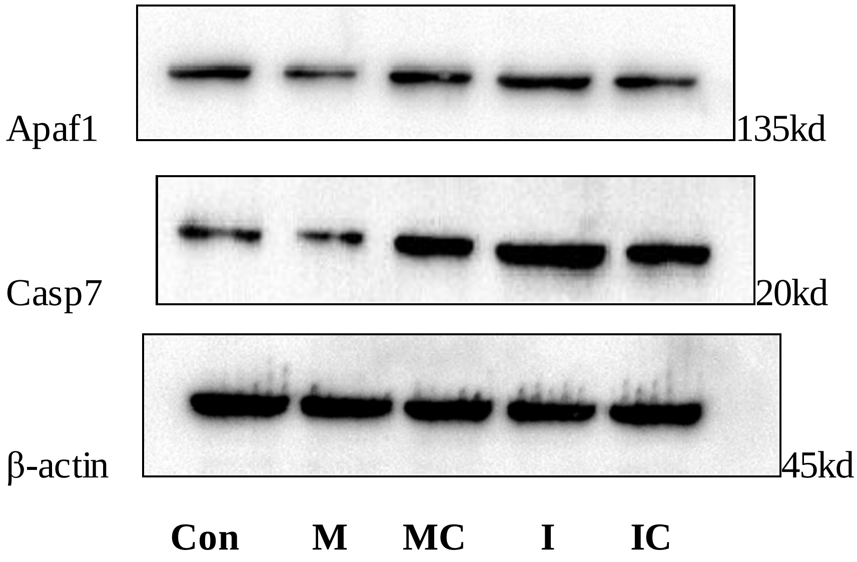

Supplementary Figure S4. Full length blots of data shown in Fig.4E

The gels were initially cut ranged from 15kd to 145 kd. . *Con* control, *M* miR-19b mimic, *MC* mimic control, *I* miR-19b inhibitor, *IC* inhibitor control.

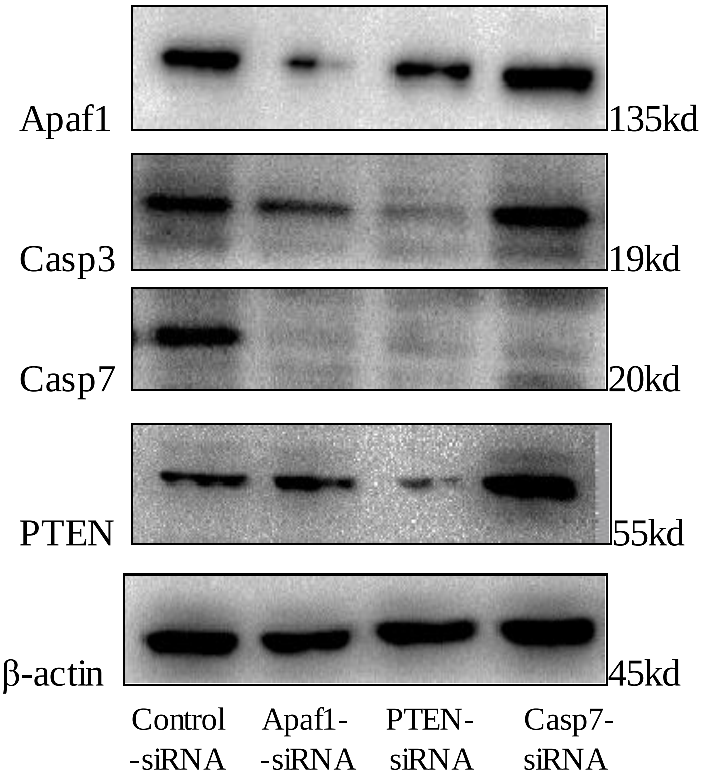

Supplementary Figure S5. Full length blots of data shown in Fig.5C

The gels were initially cut ranged from 15kd to 145 kd

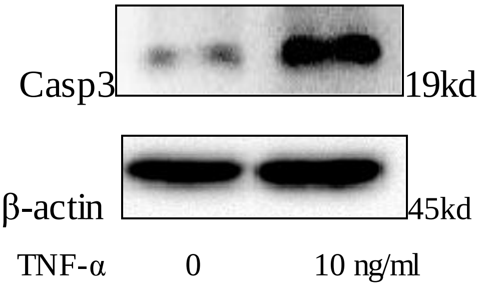

Supplementary Figure S6. Full length blots of data shown in Fig.5G

The gels were initially cut ranged from15kd to 55 kd.

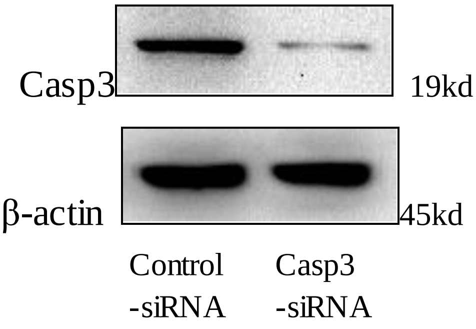

Supplementary Figure S7. Full length blots of data shown in Fig.5H

The gels were initially cut ranged from15kd to 55 kd

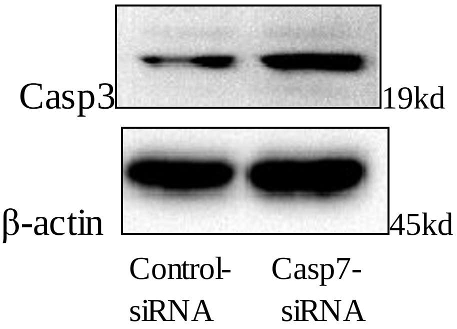

Supplementary Figure S8. Full length blots of data shown in Fig.5I

The gels were initially cut ranged from15kd to 55 kd

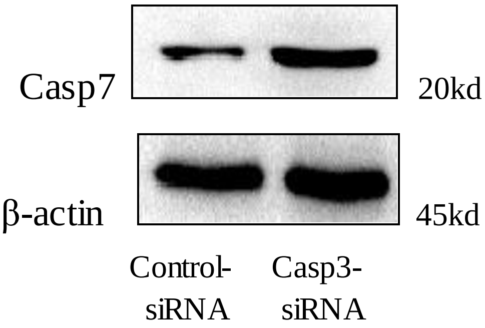

Supplementary Figure S9. Full length blots of data shown in Fig.5J

The gels were initially cut ranged from15kd to 55 kd
